# Neuropeptide‐Y Levels in ST‐Segment–Elevation Myocardial Infarction: Relationship With Coronary Microvascular Function, Heart Failure, and Mortality

**DOI:** 10.1161/JAHA.121.024850

**Published:** 2022-06-29

**Authors:** Thomas Gibbs, Nidi Tapoulal, Mayooran Shanmuganathan, Matthew K. Burrage, Alessandra Borlotti, OxAMI (Oxford Acute Myocardial Infarction) Study, Keith M. Channon, Vanessa M. Ferreira, Giovanni Luigi De Maria, Sam Dawkins, Andrew Lucking, Jeremy P. Langrish, Adrian P. Banning, Rajesh K. Kharbanda, Robin P. Choudhury, Adrian P. Banning, Robin P. Choudhury, Stefan Neubauer, Rajesh K. Kharbanda, Vanessa M. Ferreira, Keith M. Channon, Neil Herring

**Affiliations:** ^1^ Department of Physiology, Anatomy and Genetics, Burdon Sanderson Cardiac Science Centre University of Oxford United Kingdom; ^2^ Division of Cardiovascular Medicine, Radcliffe Department of Medicine, British Heart Foundation Centre of Research Excellence University of Oxford United Kingdom; ^3^ Oxford Acute Vascular Imaging Centre University of Oxford United Kingdom; ^4^ National Institute for Health Research Oxford Biomedical Research Centre Oxford University Hospitals NHS Foundation Trust Oxford United Kingdom

**Keywords:** biomarker, cardiovascular magnetic resonance imaging, microvasculature, percutaneous coronary intervention, prognosis, sympathetic cotransmitter, Autonomic Nervous System, Biomarkers, Coronary Circulation, Ischemia, Risk Factors, Heart Failure, Myocardial Infarction, Angiography, Magnetic Resonance Imaging (MRI), Prognosis, Percutaneous Coronary Intervention, Revascularization, Acute Coronary Syndromes, Coronary Artery Disease

## Abstract

**Background:**

The sympathetic cotransmitter, neuropeptide Y (NPY), is released into the coronary sinus during ST‐segment–elevation myocardial infarction and can constrict the coronary microvasculature. We sought to establish whether peripheral venous (PV) NPY levels, which are easy to obtain and measure, are associated with microvascular obstruction, myocardial recovery, and prognosis.

**Methods and Results:**

NPY levels were measured immediately after primary percutaneous coronary intervention and compared with angiographic and cardiovascular magnetic resonance indexes of microvascular function. Patients were prospectively followed up for 6.4 (interquartile range, 4.1–8.0) years. PV (n=163) and coronary sinus (n=68) NPY levels were significantly correlated (*r*=0.92; *P*<0.001) and associated with multiple coronary and imaging parameters of microvascular function and infarct size (such as coronary flow reserve, acute myocardial edema, left ventricular ejection fraction, and late gadolinium enhancement 6 months later). We therefore assessed the prognostic value of PV NPY during follow‐up, where 34 patients (20.7%) developed heart failure or died. Kaplan‐Meier survival analysis demonstrated that high PV NPY levels (>21.4 pg/mL by binary recursive partitioning) were associated with increased incidence of heart failure and mortality (hazard ratio, 3.49 [95% CI, 1.65–7.4]; *P*<0.001). This relationship was maintained after adjustment for age, cardiovascular risk factors, and previous myocardial infarction.

**Conclusions:**

Both PV and coronary sinus NPY levels correlate with microvascular function and infarct size after ST‐segment–elevation myocardial infarction. PV NPY levels are associated with the subsequent development of heart failure or mortality and may therefore be a useful prognostic marker. Further research is required to validate these findings.

**Nonstandard Abbreviations and Acronyms**
CS: coronary sinus
NPY: neuropeptide Y
PPCI: primary percutaneous coronary intervention
PV: peripheral venous

**Clinical Perspective**

**What Is New?**

- Neuropeptide‐Y levels, when measured from a peripheral vein at the time of primary percutaneous coronary intervention, correlate with coronary microvascular dysfunction, greater myocardial injury, reduced left ventricular ejection fraction 6 months after ST‐segment–elevation myocardial infarction, and subsequent heart failure and mortality over a median follow‐up of 6.4 years, even after adjustment for age and cardiovascular risk factors.
- We provide a definition of high peripheral venous neuropeptide Y that is associated with subsequent heart failure and mortality.

**What Are the Clinical Implications?**

- Neuropeptide Y, which can easily and safely be measured from a peripheral vein at a single time point after primary percutaneous coronary intervention, may be a useful biomarker to guide prognosis.
- It may also be a useful theranostic biomarker to guide the use of neuropeptide‐Y receptor antagonists given previous observations of the ability of such drugs to reduce infarct size in an animal model.

In patients with acute ST‐segment–elevation myocardial infarction (STEMI), the immediate aim is to restore coronary perfusion by expeditious revascularization of the infarct‐related epicardial vessel using primary percutaneous coronary intervention (PPCI). Despite this, one third of patients do not regain satisfactory myocardial reperfusion, experiencing a phenomenon known as “no‐reflow.” This involves an ongoing flow restriction in the microcirculation and is associated with prolonged ST‐segment elevation, larger infarct volume, lower left ventricular ejection fraction (LVEF), recurrent heart failure admission, and death.^1^ The cause for this remains unclear but is likely multifaceted.^2^ Downstream embolization of fragments of clot or thrombus into the microvasculature is thought to contribute, but during PPCI, only low volumes of these embolic particles have been observed, and clinical trials suggest intraprocedural thrombectomy to be of little benefit.^3^, ^4^ Ischemia‐reperfusion–related tissue swelling may lead to microvascular compression, direct endothelial damage, widespread platelet and neutrophil activation, and the formation of platelet plugs.^5^, ^6^ Functional vasoconstriction of the microcirculation is also emerging as a potentially important mechanism in the pathogenesis of no‐reflow and occurs in response to locally released vasoactive compounds that occur during STEMI.^7^, ^8^, ^9^

Neuropeptide Y (NPY) is a cotransmitter that is released alongside norepinephrine from sympathetic nerve terminals, particularly during conditions of sympathetic hyperactivity, such as myocardial infarction.^10^ NPY is known to be the most abundant neuropeptide in the heart and is significantly increased at the time of PPCI for STEMI, remaining high for at least 48 hours following revascularization.^11^ Clinical studies before the development of PPCI demonstrated higher peripheral levels of “NPY‐like activity” during myocardial infarction correlated with a higher incidence of mortality at 1 year.^12^ NPY is a potent vasoconstrictor, and earlier studies have shown coronary artery infusion of NPY in humans led to typical ischemic ECG changes and chest pain, without significant epicardial artery vasoconstriction.^13^ A recent study by our group suggests that this may be a result of NPY causing selective constriction of the coronary microvasculature via the Y1 receptor.^14^ This study also demonstrated that high coronary sinus (CS) NPY levels in 45 patients undergoing PPCI for STEMI correlated with increased microcirculatory dysfunction at the time of PPCI. However, CS blood sampling is challenging and requires a further invasive catheter procedure. It remains unclear whether a similar relationship exists for other measures of circulating NPY and whether this can be used to guide prognosis.

Accordingly, we examine a large cohort of patients to see whether peripheral venous (PV) NPY or the transcardiac NPY gradient (CS‐arterial difference) is closely associated with severe microvascular obstruction and reduced myocardial recovery, as seen with CS NPY. Given that the most pragmatic measurement to obtain clinically is PV NPY, we sought to ascertain whether this was associated with the development of heart failure or death in the OxAMI (Oxford Acute Myocardial Infarction) Study.

## Methods

See Data S1 Supplement for expanded methods. Local research ethics committee (REC 10/H0408/24) and institutional review board committee approval was granted, and the study complied with the Declaration of Helsinki. All study participants gave written informed consent. Patients were prospectively enrolled as part of the OxAMI Study. All data are available on reasonable request.

## Results

A total of 164 patients with STEMI were recruited and underwent PV (n=163) and/or CS and coronary arterial (n=68) blood sampling immediately after PPCI. The baseline clinical characteristics of these patients are summarized in Table 1. Most patients (76.8%) were men, with a mean age of 62.4 years, and experienced predominantly left anterior descending artery infarcts (53.7%). Overall PV and CS NPY levels were similar (20.5 [interquartile range, 10.1–34.0] versus 28.7 [interquartile range, 19.0–48.5] pg/mL) and significantly positively correlated with one another (*r*=0.92; n=67; *P*<0.001). There was no significant correlation between the CS‐A difference (−0.4 [interquartile range, −4.5 to 4.1] pg/mL) and CS NPY levels (*r*=0.08; n=68; *P*=0.52).

**Table 1:** Patient Characteristics

Baseline characteristics	PV blood sampling (n=163)	CS and aortic blood sampling (n=68)	Total (n=164)
Age, y	62.4±11.9	62.8±12.7	62.4±11.9
Men	125 (76.7)	51 (75.0)	126 (76.8)
Cardiovascular risk factors			
Previous myocardial infarction	13 (9.6)	4 (5.9)	14 (10.2)
Hypertension	73 (44.8)	27 (39.7)	74 (45.1)
Diabetes	18 (11.0)	7 (10.3)	18 (10.9)
Hypercholesterolemia	71 (43.6)	25 (36.8)	72 (43.9)
Smoking history	109 (66.9)	54 (79.4)	110 (67.1)
Family history of coronary disease	57 (35.8)	27 (42.2)	57 (35.6)
On‐admission medications			
β‐Blocker	26 (15.9)	7 (10.3)	26 (15.8)
ACE inhibitor/At II receptor blocker	34 (20.8)	10 (14.7)	34 (20.7)
Statin	36 (22.1)	13 (19.1)	36 (22.0)
Observations			
Systolic blood pressure, mm Hg	133.9±25.8	131.9±27.2	133.8±25.7
Diastolic blood pressure, mm Hg	81.4±17.6	82.4±19.4	81.4±17.5
Heart rate, bpm	77.7±19.0	79.8±19.7	77.6±18.9
Peak troponin I, mg/L	42.7±25.9	41.4±15.6	42.5±26.0
Pain‐to‐balloon time, min	174.0 (120.0–282.0)	180.0 (120.0–313.5)	174.5 (120.0–279.0)
Infarct artery			
LAD	86 (53.4)	55 (82.1)	87 (53.7)
LCx/Int	20 (12.3)	12 (17.9)	20 (12.2)
RCA	56 (34.6)	0 (0)	56 (34.4)

Values are mean±SD, number (percentage), or median (interquartile range). ACE indicates angiotensin‐converting enzyme; At II, angiotensin II; bpm, beats per minute; CS, coronary sinus; Int, intermediate artery; LAD, left anterior descending artery; LCx, left circumflex artery; PV, peripheral venous; and RCA, right coronary artery.

### Correlations of PV, CS, and Transcardiac Gradient NPY Levels With Invasive and Imaging Measures of Microvascular Function and Imaging Measures of Left Ventricular Functional Recovery

Like CS NPY levels, PV NPY levels (but not a CS‐A difference) correlated significantly with a lower coronary flow reserve measured via coronary flow wire (Table 2). PV NPY levels also correlated with several cardiac magnetic resonance imaging parameters of myocardial injury and subsequent recovery following PPCI, as observed with CS NPY levels. These include a significant positive correlation with the extent of myocardial edema observed at 2 days, a positive correlation with late gadolinium enhancement (LGE) extent, and inverse correlation with LVEF 6 months after PPCI for STEMI, as illustrated in Table 2. No significant correlations were observed for the CS‐A difference in NPY levels for any invasive or imaging parameters.

**Table 2:** Correlations Between Arterial NPY Levels, Coronary Hemodynamics, and Cardiac Magnetic Resonance Measurements

Variable	CS (n=68)		PV (n=163)		CS‐A (n=68)	
	*R*	*P* value	*R*	*P* value	*R*	*P* value
Coronary hemodynamics						
Coronary flow reserve	−0.24	<0.05*	−0.23	<0.01*	−0.13	0.30
Index of microcirculatory resistance	0.09	0.50	0.03	0.72	−0.14	0.26
Post‐PPCI cardiac MRI						
Ejection fraction	−0.18	0.22	−0.26	0.01*	−0.06	0.66
Microvascular obstruction	0.49	<0.001*	0.09	0.42	−0.14	0.35
Edema (% LV)	0.49	<0.001*	0.25	0.02*	−0.03	0.82
Late gadolinium enhancement	0.36	0.01*	0.07	0.52	−0.12	0.41
End‐diastolic volume	0.12	0.42	0.12	0.23	0.09	0.53
End‐systolic volume	0.15	0.35	0.20	0.05*	0.18	0.28
6‐mo Cardiac MRI						
Ejection fraction	−0.43	0.01*	−0.26	0.02*	0.29	0.08
End‐diastolic volume	0.08	0.61	0.09	0.43	−0.07	0.69
End‐systolic volume	0.23	0.16	0.23	0.03*	−0.19	0.25
Late gadolinium enhancement	0.57	<0.001*	0.27	0.01*	−0.11	0.52

CS indicates coronary sinus; LV, left ventricle; MRI, magnetic resonance imaging; NPY, neuropeptide Y; PPCI, primary percutaneous coronary intervention; and PV, peripheral venous.

* indicates statistical significance.

### Survival Analysis

Patients were followed up for a period of 6.4 (interquartile range, 4.1–8.0) years following the index event. During follow‐up, 20 patients (12.2%) developed heart failure, 20 patients (12.2%) died, and 34 patients (20.7%) in total experienced events, reaching the composite primary end point of heart failure or mortality. Patients in the events group were more likely to experience hypertension, diabetes, or hypercholesterolemia, and admission heart rate was significantly higher (Table 3). TIMI (Thrombolysis in Myocardial Infarction) flow at presentation and pain‐to‐balloon time were similar in both groups. Coronary flow reserve was significantly lower in those experiencing events, and index of microcirculatory resistance was higher (Table 3). The cardiovascular magnetic resonance scan at 6 months, however, revealed significantly larger infarct size (as measured by LGE extent) in patients experiencing heart failure or death, as well as a trend toward a lower LVEF. PV NPY was significantly higher in those patients who sustained adverse events compared with those who did not (28.6 versus 18.5 pg/mL; *P*=0.03) (Table 3).

**Table 3:** Patient Characteristics by Clinical Outcome

Baseline characteristics	Heart failure/mortality (n=34)	Event‐free survival (n=130)	*P* value
Age, y	71.3±10.5	60.1±11.2	<0.000001*
Men	27 (79.4)	99 (76.2)	0.69
Cardiovascular risk factors			
Hypertension	22 (64.7)	52 (40)	0.01*
Diabetes	11 (32.4)	7 (5.4)	<0.00001*
Hypercholesterolemia	21 (61.8)	51 (39.2)	0.02*
Smoking history	21 (61.8)	89 (68.5)	0.46
Previous myocardial infarction	4 (12.9)	10 (9.5)	0.59
Family history of coronary disease	11 (32.4)	46 (36.5)	0.65
Observations			
Systolic blood pressure, mm Hg	140.3±24.1	132.2±26.0	0.11
Diastolic blood pressure, mm Hg	80.6±14.0	81.6±18.3	0.73
Heart rate, bpm	88 (72–100)	71 (63–84.3)	<0.01*
Peak troponin I, mg/L	50 (43.6–50)	50 (25.4–50)	0.16
Pain‐to‐balloon time, min	175 (130–300)	174 (120–268)	0.53
Infarct artery			
LAD	20 (58.8)	67 (52.3)	0.50
LCx/Int	7 (20.6)	13 (10.1)	0.10
RCA	7 (20.6)	49 (38.0)	0.06
TIMI flow at presentation			
0	26 (86.7)	80 (72.7)	0.21
1	1 (3.2)	9 (8.2)	0.34
2	1 (3.2)	14 (12.7)	0.13
3	3 (9.7)	6 (5.4)	0.61
Coronary hemodynamics			
CFR	1.3 (1.0–1.6)	1.6 (1.2–2.2)	0.01*
IMR	39.6 (27.9–96.4)	26.4 (18.0–42.0)	<0.01*
Cardiac MRI			
MVO, %	1.3 (0–6.3)	1.0 (0–3.8)	0.66
Ejection fraction at 48 h, %	45.3±11.4	48.4±8.9	0.22
LGE at 48 h, %	30.6±19.5	40.0±13.5	0.95
Edema, %	36.8±20.2	42.7±13.0	0.28
Ejection fraction at 6 mo, %	49.0 (43.3–58.5)	57 (47.5–61.3)	0.06
LGE at 6 mo, %	29.0±13.2	19.0±12.3	<0.01*
Peripheral venous NPY, pg/mL	28.6 (13.5–49.0)	18.5 (9.5–32.2)	0.03*

Values are mean±SD, number (percentage), or median (interquartile range). Bpm indicates beats per minute; CFR, coronary flow reserve; IMR, index of microvascular resistance; Int, intermediate artery; LAD, left anterior descending artery; LCx, left circumflex artery; LGE, late gadolinium enhancement; MRI, magnetic resonance imaging; MVO, microvascular obstruction; NPY, neuropeptide Y; RCA, right coronary artery; and TIMI, thrombolysis in myocardial infarction.

* inidicates statistical significance.

PV NPY as a continuous variable was associated with heart failure or death, with an estimated hazard ratio (HR) of 1.014 (95% CI, 1.006–1.022; *P*<0.001). A multivariable Cox proportional hazard model, adjusting for age, sex, smoking, hypertension, hypercholesterolemia, diabetes, family history of cardiovascular disease, and previous myocardial infarction, did not affect the association of PV NPY with the combined end point (HR, 1.011 [95% CI, 1.001–1.021]; *P*=0.03). We then used binary recursive partitioning analysis to define a PV NPY threshold that best identifies patients reaching the primary outcome. This cutoff (21.4 pg/mL) had a C statistic of 0.62 (95% CI, 0.52–0.73; *P*=0.03) for the combined end point (heart failure or death), whereas heart failure diagnosis as an independent end point had a C statistic of 0.67 (95% CI, 0.56–0.79; *P*=0.01). Patients with high PV NPY were older, but otherwise the 2 groups were well matched in terms of cardiovascular risk factors (Table 4). Patients with high PV NPY had similar pain‐to‐balloon times and TIMI flow at presentation, but lower coronary flow reserve and a trend toward greater myocardial edema. Cardiovascular magnetic resonance imaging in patients with high PV NPY revealed a significantly higher percentage of LGE and lower LVEF at 6 months. Kaplan‐Meier survival analysis demonstrated that high PV NPY levels are associated with an increased incidence of death (HR, 3.07 [95% CI, 1.18–7.97]; *P*=0.02), heart failure (HR, 6.16 [95% CI, 2.04–18.6]; *P*<0.001), or both (HR, 3.49 [95% CI, 1.65–7.4]; *P*<0.001) (Figure – Panels A, C, and E). Adjusting for age, sex, smoking, hypertension, hypercholesterolemia, diabetes, family history of cardiovascular disease, and previous myocardial infarction did not affect the association of high PV NPY with the combined end point (HR, 3.31 [95% CI, 1.28–8.53]; *P*=0.01) or heart failure (HR, 30.1 [95% CI, 3.23–280.42]; *P*=0.003), but PV NPY was not a significant independent risk factor for death alone following adjustment (*P*=0.66), as shown in Figure – Panels B, D, and F.

**Table 4:** Patient Characteristics by High Versus Low PV NPY Levels

Baseline characteristics	High NPY (≥21.4 pg/mL; n=78)	Low NPY (<21.4 pg/mL; n=85)	*P* value
Age, y	65.8±12.4	59.2±10.7	<0.001*
Men	55 (70.5)	70 (82.4)	0.08
Cardiovascular risk factors			
Hypertension	40 (51.2)	33 (38.9)	0.11
Diabetes	8 (10.3)	10 (11.8)	0.76
Hypercholesterolemia	37 (47.4)	34 (40.0)	0.34
Smoking history	53 (67.9)	56 (65.9)	0.78
Previous myocardial infarction	6 (9.0)	7 (10.3)	0.79
Family history of coronary disease	27 (36.0)	30 (35.7)	0.97
Observations			
Systolic blood pressure, mm Hg	136.4±26.8	131.7±24.7	0.25
Diastolic blood pressure, mm Hg	81.9±18.7	81.0±16.5	0.77
Heart rate, bpm	78 (63–90)	72 (66–85)	0.57
Peak troponin I, mg/L	50 (38.7–50)	50 (21.9–50)	0.31
Pain‐to‐balloon time, min	166 (120–270)	176 (121.8–282.5)	0.61
Infarct artery			
LAD	48 (63.2)	38 (44.7)	0.02*
LCx/Int	10 (13.0)	10 (11.7)	0.81
RCA	19 (24.6)	37 (43.5)	0.01*
TIMI flow at presentation			
0	44 (75.9)	62 (74.7)	0.88
1	4 (6.9)	6 (7.2)	0.94
2	7 (12)	8 (9.6)	0.65
3	3 (5.2)	7 (8.4)	0.46
Coronary hemodynamics			
CFR	1.3 (1.1–1.9)	1.7 (1.3–2.2)	0.04*
IMR	31 (20.2–52.2)	29.6 (18.9–43.5)	0.54
Cardiac MRI			
MVO, %	1 (0–5.2)	1 (0–3.5)	0.30
Ejection fraction at 48 h, %	46.0±10.0	49.3±8.6	0.09
LGE at 48 h, %	32.7±14.9	29.9±13.9	0.35
Edema, %	44.8±13.9	39.6±14.6	0.08
Ejection fraction at 6 mo, %	53 (43–57)	58 (50–64)	0.01*
LGE at 6 mo, %	24.3±13.4	17.7±11.9	0.02*

Values are mean±SD, number (percentage), or median (interquartile range). Bpm indicates beats per minute; CFR, coronary flow reserve; IMR, index of microvascular resistance; Int, intermediate artery; LAD, left anterior descending artery; LCx, left circumflex artery; LGE, late gadolinium enhancement; MRI, magnetic resonance imaging; MVO, microvascular obstruction; NPY, neuropeptide Y; PV, peripheral venous; RCA, right coronary artery; and TIMI, thrombolysis in myocardial infarction.

* indicates statistical significance.

**Figure 1:**
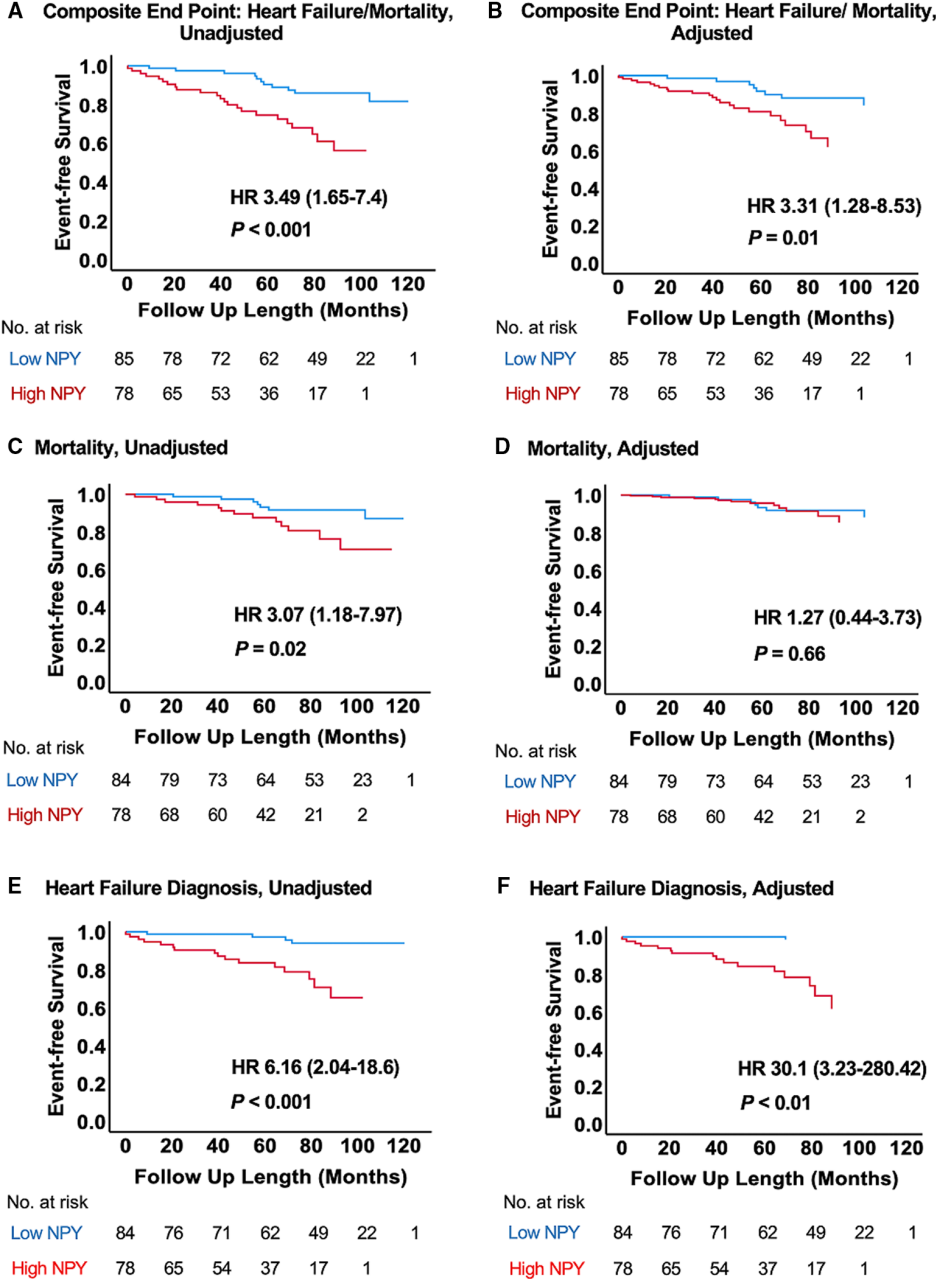
Kaplan‐Meier survival analysis, illustrating event‐free survival following ST‐segment–elevation myocardial infarction, according to peripheral venous neuropeptide‐Y (NPY) levels before (A, C, and E) and after (B, D, and F) adjustment for age, sex, hypertension, diabetes, hypercholesterolemia, family history of coronary artery disease, smoking history, and previous myocardial infarction. Binary recursive partitioning analysis was used to derive a cutoff for high and low NPY (≥21.4 and <21.4 pg/mL, respectively). **A** and **B**, Events are of a composite of death and heart failure diagnosis (n=34). **C** and **D**, Events are all‐cause mortality (n=20). **E** and **F**, Events are heart failure diagnosis (n=20). HR indicates hazard ratio.

## Discussion

In this study, we show that high peripheral venous NPY levels, analyzed immediately after PPCI, correlate with coronary microvascular dysfunction, greater myocardial injury, and reduced LVEF 6 months after STEMI. Moreover, high PV NPY levels are associated with subsequent heart failure and mortality, even after adjustment for age and cardiovascular risk factors.

These findings build on our previous observations in a small translational study, where we showed that CS NPY levels are elevated during STEMI and are associated with increased microvascular dysfunction. The present study confirms that CS NPY has the strongest relationship with indexes of reperfusion and left ventricular functional recovery, probably because it more accurately reflects local cardiac NPY levels that the coronary microcirculation is exposed to. PV NPY levels also correlate well with these parameters, despite additional contributions from hepatic and mesenteric release.^15^ Interestingly, there was no significant relationship between indexes of reperfusion and the transcardiac NPY gradient, which was small in magnitude. Peripheral and cardiac NPY levels are extremely high during STEMI, and it is likely that they will have equilibrated by the time of PPCI, often several hours from the onset of chest pain and heightened sympathetic drive.

We have recently shown that CS NPY levels are associated with adverse clinical outcomes in patients with stable chronic heart failure who are undergoing implantation of cardiac resynchronization devices.^16^ However, CS NPY is not practical to obtain routinely, and requires a second central venous cannulation procedure that introduces additional risk. In contrast, NPY can be measured easily and safely from a peripheral vein, and the major finding of the present study is that high levels of PV NPY are associated with the development of heart failure or death after reperfusion. This relationship is maintained even after adjustment for age, sex, and major cardiovascular risk factors, including hypertension, diabetes, high cholesterol, family history, smoking status, and previous myocardial infarction. Thus, a simple and readily accessible PV NPY measurement is a potentially useful biomarker in this cohort that could offer incremental information on other known prognosticators. Indeed, NPY at the time of PPCI for STEMI correlates strongly with infarct size, as measured by LGE and ejection fraction at 6 months, and has a similar relationship with events on univariable and multivariable analysis.

NPY has previously been shown to induce myocardial ischemia, demonstrable through ECG ST‐T wave changes, reduction of intramyocardial pH, and LVEF in dogs.^17^ Exogenous administration of NPY in humans with microvascular angina induced transient myocardial ischemia despite minimal vasospasm of the epicardial coronary arteries.^18^ We have shown that NPY, which has a long plasma half‐life, is significantly elevated during PPCI for STEMI and remains high for at least 48 hours.^11^ In rats, we have explored the mechanism by which NPY constricts the microcirculation via a Y1 receptor pathway and demonstrated that this receptor is also expressed on vascular smooth muscle cells in the media of human coronary microarteries.^14^ The Y1 receptor is also expressed on ventricular myocytes and can lead to calcium loading and increased propensity to arrhythmia, even in the presence of β‐blockade.^19^ In a rat model of STEMI, we have provided proof of principle that antagonism of the Y1 receptor can reduce infarct size and the incidence of ventricular arrhythmia after subsequent reperfusion.^14^, ^19^

Also, there are other ways in which NPY plays an important mechanistic role in the pathophysiology of atherosclerosis, STEMI, and ischemic heart failure. For example, genetic polymorphisms in the NPY gene and those of several of its receptors are associated with early‐onset atherosclerosis.^10^ NPY can be taken up into megakaryocytes; and following plaque rupture, activated platelets may release NPY locally. Although in the short‐term, NPY may cause microvascular constriction, in the longer‐term, an elevation in dipeptidyl peptidase‐4 expression within the endothelium may increase cleavage of NPY_1–36_ to NPY_3–36_, which has a higher binding affinity for Y2 and Y5 receptors, promoting angiogenesis. Dipeptidyl peptidase‐4 inhibitors used to treat type 2 diabetes are associated with a significant increase in the risk of serious heart failure events in several large clinical trials.^20^ In the short‐term, NPY may reduce vagal acetylcholine release via the Y2 receptor and directly maintain cardiac contraction and inotropy, but in the longer‐term, it promotes ventricular myocyte hypertrophy.^10^, ^21^, ^22^ It is interesting to note that with our data, the relationship between high PV NPY and heart failure or mortality is lost during multivariable Cox regression analysis after adjustment for coronary flow reserve, LGE extent, and LVEF at 6 months (*P*=0.41). Mechanistically, this is consistent with the hypothesis that NPY contributes to microvascular dysfunction and subsequent infarct size, leading to heart failure and mortality following STEMI, rather than having other independent effects outside that of influencing infarct size.

Measuring PV NPY levels may identify patients in whom closer monitoring and more aggressive interventions are required. It is cheaper and more practical to measure PV NPY than routinely perform coronary flow wire measurements of microvascular function or undertake cardiovascular magnetic resonance at both 2 days and 6 months following the event, which also offer valuable prognostic information but are not readily available in all centers.^23^, ^24^, ^25^, ^26^ In addition to standard pharmacotherapy, antagonizing NPY Y1 receptors may also have the potential to mitigate the effects of no‐reflow following STEMI, leading to improved outcomes in selected patients with high initial PV NPY. In this way, high PV NPY has the potential to also be a theranostic biomarker.

### Study Limitations

Conceivably, CS NPY could more strongly correlate with events, given its stronger association with microvascular resistance, and a further study powered to evaluate this association would be informative. In addition to this, more precise temporal dynamics of circulating NPY following PPCI for STEMI may be more strongly correlated with poorer outcomes, although this would be more challenging to obtain clinically compared with a test at a single time point when NPY levels are at their highest.

## Conclusions

Both PV and CS NPY levels significantly correlate with microvascular function and infarct size after STEMI, whereas the CS‐A difference in NPY levels does not, presumably as both cardiac and peripheral NPY release is extremely high and equilibrated by the time of PPCI. High PV NPY levels, which are easy to obtain and measure following coronary intervention, are independently associated with subsequent heart failure and death and could prove to be a useful biomarker in risk stratifying these patients.

## Appendix

### OxAMI (Oxford Acute Myocardial Infarction) Study Investigators

Robin P. Choudhury, DM; Rajesh K. Kharbanda, MBChB, PhD; Adrian P. Banning, MBBS, MD; Jeremy P. Langrish, MB BCh, PhD; Andrew Lucking, MBChB, PhD; Sam Dawkins, MBBS, DPhil; Giovanni Luigi De Maria, MD, PhD; Vanessa M. Ferreira, MD, DPhil; Keith M. Channon, MD, PhD.

## Sources of Funding

The study was supported by a British Heart Foundation Senior Clinical Research Fellowship (FS/SCRF/20/32005) to Dr Herring, a BHF Chair award (CH/16/1/32013) to Dr Channon, the BHF Oxford Centre of Research Excellence (RE/13/1/30181), and the National Institute for Health Research Oxford Biomedical Research Centre.

## Disclosures

None.

## Supporting information

Data S1
References 11, 19, 27, 28, 29, 30, 31
