# Supplementary material for: Neuropeptide‐Y Levels in ST‐Segment–Elevation Myocardial Infarction: Relationship With Coronary Microvascular Function, Heart Failure, and Mortality
Source: J Am Heart Assoc. 2022 Jun 29;11(13):e024850. doi: 10.1161/JAHA.121.024850 (PMC9333365; doi:10.1161/JAHA.121.024850)
Supplement: Supplementary file 1 — Data S1 References 11, 19, 27, 28, 29, 30, 31 [file JAH3-11-e024850-s001.pdf]

SUPPLEMENTAL MATERIAL

Data S1. Supplemental Methods

164 patients who underwent PPCI for STEMI [27] at Oxford University Hospitals NHS Foundation Trust were prospectively enrolled as part of the Oxford Acute Myocardial Infarction (OxAMI) study. Patients were excluded if they suffered symptom duration greater than 12 hours, cardiogenic shock or had contraindications to cardiac magnetic resonance imaging. PPCI was performed according to current international guidelines [28]. Verbal consent was obtained at the time of emergency PPCI and all patients gave written consent before any data or samples were analyzed. Local research ethics committee approval was granted (REC 10/H0408/24) and the study complied with the Declaration of Helsinki.

Pressure wire assessment of coronary microcirculation

Immediately after balloon angioplasty/stent insertion, a pressure-wire (Certus, Abbot) was advanced into the distal third of the infarct-related artery. This allowed for invasive assessment of coronary flow reserve (CFR) and index of microcirculatory resistance (IMR) to be measured, as previously described [11].

Blood sampling measurements

Peripheral venous blood samples were taken immediately following PPCI. Coronary sinus blood samples were obtained by cannulation with a 6F catheter via the right femoral or antecubital vein. Arterial blood samples were taken via the coronary guide catheter at the aortic root. Where they were all measured, arterial, CS and peripheral venous samples were taken sequentially and typically within a few seconds of each other. Blood was collected into standard tubes containing dipotassium ethylenedinitrotetraacetic (EDTA) acid and subsequently centrifuged for 12 minutes at 1300rcf before aliquoting into 200 μl vials and stored at -80°C until the time of assay. A commercially available ELISA kit (EZHNPY-25K,

Millipore, USA) was used according to the manufacturer's instructions to measure NPY concentration, with a lower limit of detection of 3 pg/ml as described previously [19]. CS NPY samples were recorded from patients suffering left coronary infarcts only, as venous drainage from the right coronary is also via the thebesian system [29].

Cardiovascular magnetic resonance imaging (CMR)

CMR was performed using standardized acquisition protocols using a 3 T CMR system (either MAGNETOM TIM-Trio or MAGNETOM Verio, Siemens) and was carried out at 2 days and at 6 months following PPCI. As described previously, Steady State Free Precession cine imaging, T1 and T2-prepared and late gadolinium enhancement (LGE) imaging were used to assess left ventricular function, area at risk, infarct size and microvascular obstruction (MVO) [30].

Outcome measures and follow-up

Patients were followed up from the point of their PPCI admission. This was done via Oxford University Hospital's electronic patient records system, by contacting the patient's GP surgery, and/or by contacting the patient themselves. The primary end-point was a composite of mortality and heart failure diagnosis. Secondary end-points were mortality and heart failure diagnosis in isolation. Heart failure diagnosis was defined in accordance with ESC guidelines as any new congestive heart failure symptoms or signs following the index event, supported by imaging evidence of left ventricular dysfunction or raised levels of natriuretic peptide [31]. Heart failure diagnoses were adjudicated by two physicians independently.

Statistical analysis

Continuous variables are expressed as mean±standard deviation, or as median [interquartile range] for non-normally distributed data. Categorical data values are expressed as frequencies and percentages. A Pearson correlation coefficient was used to measure linear correlation between two normally distributed variables and a Spearman's coefficient for non-parametric data. An unpaired student's *t*-test was used to compare mean values of continuous data between two independent groups. A Mann-Whitney *U* test was used for group-wise comparison of non-parametric data. Binary recursive partitioning analysis (BPRA) was used to generate the most discriminative cut-off value of high vs low NPY with regards to the composite endpoint (death or heart failure diagnosis) for Kaplan-Meier (KM) survival analysis. The log-rank was used to assess the difference between survival distributions. KM curves were generated to display the proportion of patients above and below optimal BPRA thresholds surviving at a given time. Univariable and multivariable Cox proportional-hazards models were fitted to assess survival by estimating hazard ratios (HRs) for the combined and individual endpoints after adjustment for potential confounders. All data was analyzed using Microsoft Excel and IBM SPSS Statistics version 25 for Macintosh, with two-tail values of $P < 0.05$ accepted as statistically significant.
